# Unintegrated HIV-1 DNA recruits cGAS via its histone-binding domain to escape innate immunity

**DOI:** 10.1073/pnas.2424465122

**Published:** 2025-03-11

**Authors:** Cyprien Jahan, Lucie Bonnet-Madin, Shinichi Machida, Bijan Sobhian, Suzie Thenin-Houssier, Monsef Benkirane

**Affiliations:** ^a^Institut de Génétique Humaine, Laboratoire de Virologie Moléculaire, CNRS Université de Montpellier-UMR9002, Montpellier 34000, France; ^b^Department of Structural Virology, National Center for Global Health and Medicine, Tokyo 162-8655, Japan

**Keywords:** HIV, chromatin, innate immunity, cGAS

## Abstract

To ensure optimal replication and spread, viruses have evolved countermeasures to evade type 1 IFN-mediated antiviral activity. During the early viral replication cycle steps until uncoating, the HIV-1 core protects viral pathogen associated molecular patterns (viral RNA and reverse transcription products) from recognition by innate immune sensors, including cGAS. However, after capsid uncoating, unintegrated viral DNA (uvDNA) becomes accessible. Here, we show that HIV-1 uses chromatin-mediated cGAS inactivation as a mechanism to protect its uvDNA from innate immune activation.

Innate immunity provides an important first line of defense against invading pathogens or harmful injury and relies on pattern recognition receptors expressed on the surface of or within immune cells to recognize pathogen-associated molecular patterns. The cyclic GMP-AMP synthase (cGAS)–stimulator of interferon genes (STING) DNA pathway has emerged as a key innate immune pathway important for antiviral immunity. cGAS binds to double-stranded DNA and catalyzes the production of the second messenger 2′3′-cyclic GMP-AMP, a diffusible cyclic dinucleotide that activates the endoplasmic adaptor protein STING. Activated STING then functions as a platform to recruit and activate the kinase TBK1, which phosphorylates and activates the transcription factor IRF3, leading to the induction of type I interferon (IFN) production (1). DNA sensors were initially proposed to be sequestered in the cytoplasm to avoid autoreactivity, with self-DNA protected by the nuclear membrane. However, recent studies revealed that cGAS is abundant in the nucleus, where it is tightly tethered to chromatin by binding to the acidic patch formed by histones H2A and H2B in the nucleosome core. Importantly, when bound to nucleosomes, the catalytic domain of cGAS is buried, and cGAS is locked into a monomeric state, which prevents its dimerization and activation (2, 3).

Upon entry into the host cell, HIV-1 RNA genome (vRNA) is transformed into double-stranded viral DNA (vDNA). vDNA is first loaded with histones and then integrated into the host genome, where it becomes indistinguishable from chromosomal DNA. By protecting vRNA and vDNA from recognition by both RNA and DNA sensors, the viral core plays a key role in innate immune escape (4–8). However, how vDNA evades cGAS recognition after uncoating and before integration is unknown.

In this study, we reveal a mechanism of HIV-1-mediated prevention of cGAS activation involving the uvDNA chromatin structure.

## Results and Discussion

### uHIV-1 DNA Sensing in Primary CD4+ T Cells in the Absence of POLE3 Involves the Sensor cGAS.

**Fig. 1.**
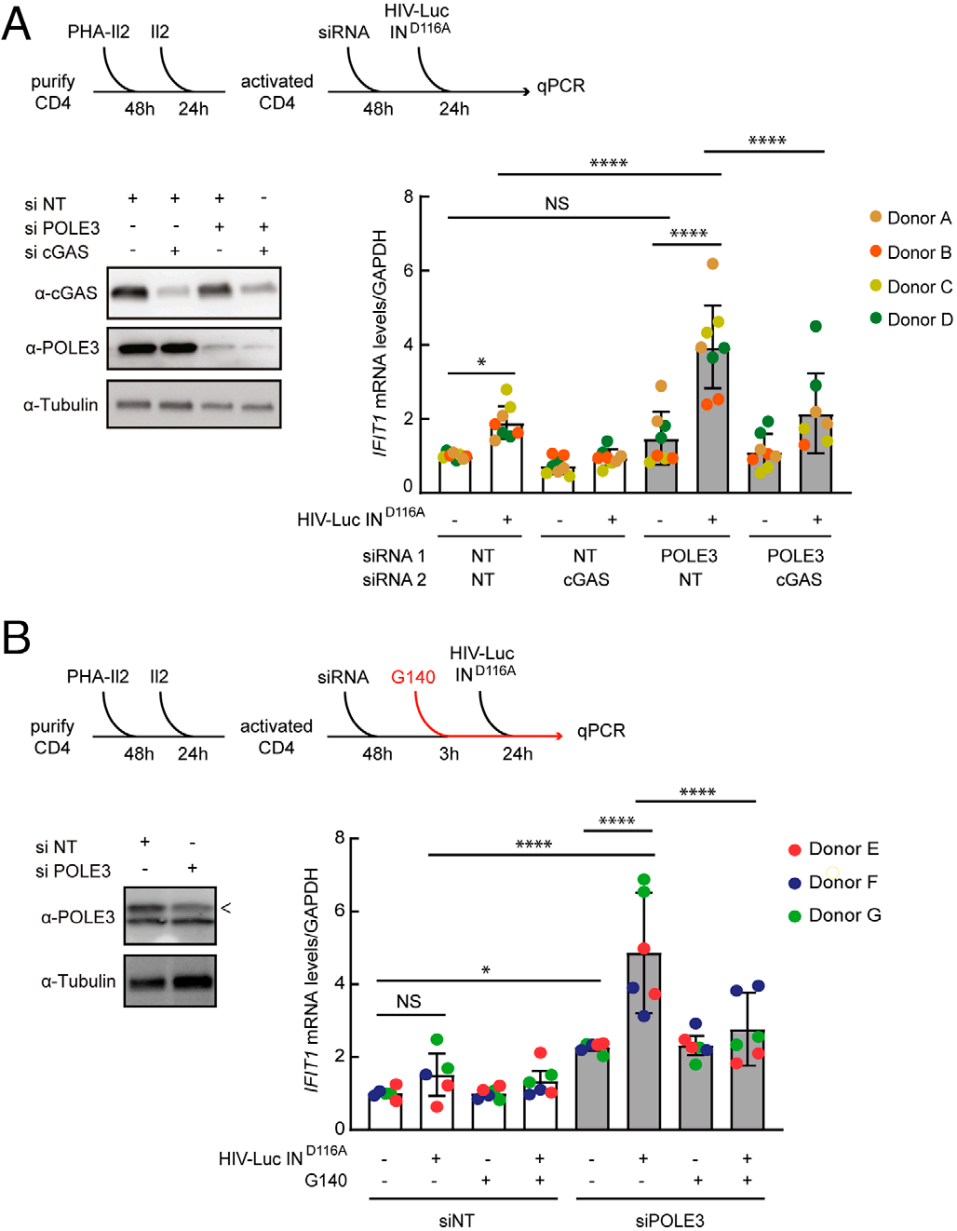
POLE3 knockdown results in cGAS sensing of unintegrated HIV-1 DNA. (*A*) Experimental scheme: primary CD4^+^ T cells isolated from four healthy donors were activated with PHA/IL2, electroporated with the indicated siRNA, and infected with VSV-G- HIV-Luc IN^D116A^ in duplicate (*Upper* panel). Representative immunoblot of POLE3 and cGAS expression (*Left* panel). *IFIT1* mRNA expression (normalized to GAPDH mRNA expression) was measured by qPCR at 24 hpi (*Right* panel). (*B*) Experiment performed as described in (*A*), except that NT and POLE3 siRNA-transfected CD4+ T cells were treated with DMSO or the cGAS inhibitor (G140) 3 h before infection with VSV-G- HIV-Luc IN^D116A^. *IFIT1* mRNA expression was quantified as described in *A*. *P < 0.05, **P < 0.01, ***P < 0.001, ****P < 0.0001; Bonferroni’s multiple comparisons test (ANOVA test).

We previously showed that POLE3 knockdown results in HIV-1 DNA sensing and innate immune response activation in primary CD4^+^ T cells (9). To determine the role of cGAS in this process, primary CD4^+^ T cells were activated with phytohemagglutinin/Interleukin-2 (PHA-Il2), transfected with nontargeting (NT) or POLE3 siRNA in combination with cGAS siRNA, and subsequently infected with a VSV-G-pseudotyped HIV-Luc virus harboring a mutation in the integrase catalytic domain (HIV-Luc IN^D116A^) (Fig. 1*A* and Dataset S1). Efficient POLE3 and cGAS KD was observed (Fig. 1*A*). The expression of the IFN-stimulated gene *IFIT1* was quantified. As previously reported, HIV-Luc IN^D116A^ infection of NT siRNA control cells resulted in weak but significant induction of *IFIT1* compared with that in uninfected control cells, whereas HIV-Luc IN^D116A^ infection of POLE3 KD cells resulted in a fourfold increase in *IFIT1* mRNA expression. *IFIT1* induction was significantly decreased when both cGAS and POLE3 were depleted (Fig. 1*A*). Next, primary CD4^+^ T cells were transfected with NT or POLE3 siRNA, treated with the cGAS inhibitor, G140, or DMSO for 3 h, and subsequently infected with HIV-Luc IN^D116A^ for 24 h (Fig. 1*B* and Dataset S2). POLE3 KD resulted in a fivefold increase in *IFIT1* expression compared with that in noninfected cells (Fig. 1*B*). This induction was significantly decreased in G140-treated POLE3 KD CD4^+^ T cells (Fig. 1*B*), suggesting that G140 inhibits the POLE3-dependent response triggered by uHIV-1 DNA. Collectively, these results showed that cGAS is involved in uHIV-1 DNA sensing in primary CD4^+^ T cells in the absence of POLE3. However, we cannot exclude that cGAS activation may result in an induction of host factor with antiviral activity targeting uHIV-1 DNA.

### HIV-1 DNA-Mediated cGAS Activation in POLE3 KO Cells Reduces Viral Replication.

**Fig. 2.**
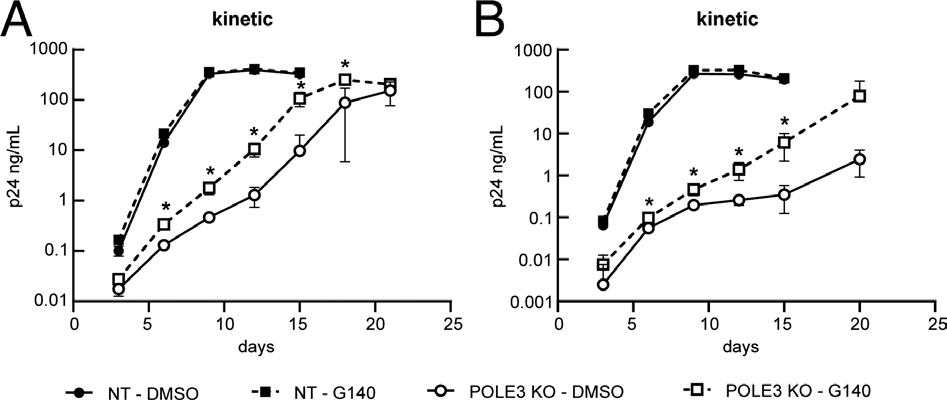
HIV-1 DNA-mediated cGAS activation in POLE3 KO cells reduces viral replication. HIV-1 replication kinetics in POLE3 KO HeLa-P4 (white dots) and NT control (black dots) cells treated with the cGAS inhibitor G140 (squares and dashed lines) or DMSO (circles and continuous lines) were measured by quantifying p24 antigen in the culture supernatant every 3 d post infection. Each kinetic plot represents 2 independent experiments with duplicates. *P < 0.05, independent Student’s *t* test

Studies aimed at understanding uHIV-1 DNA transcriptional regulation revealed host factors responsible for its repression (9–11). We reported that POLE3 maintained uHIV-1 DNA in a repressed chromatin state. Surprisingly, POLE3 depletion dramatically impaired HIV-1 replication, an effect explained by reduced vDNA integration and innate immune activation (9). To assess the contribution of cGAS-mediated innate immune response to the reduced viral replication observed in the absence of POLE3, we analyzed the impact of cGAS inhibition on viral replication in HeLa-P4 POLE3 knockout (KO) and control cells. HeLa-P4 were pretreated with G140 (10 µM) or DMSO 3 h before infection with a replication-competent virus (NL4.3). POLE3 depletion dramatically impaired viral replication compared with that in control cells (9) (Fig. 2). G140 did not impact HIV-1 replication in control cells but partially restored virus replication in POLE3 KO cells (Fig. 2). This result shows that in addition to defect in viral integration, cGAS-mediated antiviral activity contributes to the observed viral replication impairment in the absence of POLE3. However, this experiment does not exclude that other DNA sensors may also be involved. Further experiments are needed to determine whether the observed cGAS-mediated immune activation involves the STING/TBK1/IRF3 pathway.

### cGAS Is Loaded Onto uHIV-1 DNA via Its Histone-Binding Domain.

**Fig. 3.**
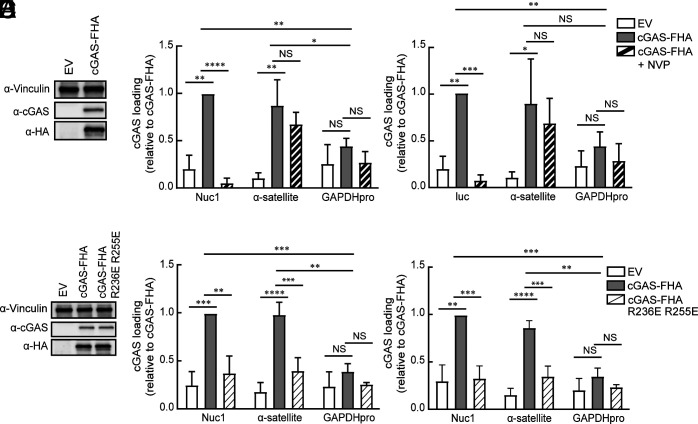
uHIV-1 DNA tethers cGAS via chromatin binding. (*A*) Western blot analysis of HeLa KO cells stably transduced with Flag-HA EV or cGAS-FHA with the indicated antibodies. (*B* and *C*) ChIP analysis of cGAS binding to uHIV-1 DNA. HeLa-EV and HeLa-cGAS-FHA were infected with VSV-G- HIV-Luc IN^D116A^ with or without NVP treatment. Chromatin was prepared 24 hpi. ChIP was performed with an α-HA antibody. qPCR analysis was performed with the indicated primers. Data are presented relative to cGAS-FHA with HIV-specific primers: Nuc1 (*B*) or luc (*C*). Mean ± SD values of four independent experiments. (*D*) Western blot analysis of HeLa KO cells stably transduced with EV, cGAS-FHA, or the cGAS mutant (cGAS-FHA R236E R255E) using the indicated antibodies. (*E* and *F*) ChIP analysis of cGAS binding to histones H2A-H2B on uHIV-1 DNA. HeLa-EV, HeLa-cGAS-FHA, and HeLa-cGAS-FHA R236E R255E cells were infected with VSV-G- HIV-Luc IN^D116A^. ChIP was performed as described in *B* and *C*. Data are presented relative to cGAS-FHA with HIV-specific primers: Nuc1 (*E*) or luc (*F*). Mean ± SD values of five independent experiments. *P < 0.05, **P < 0.01, ***P < 0.001, ****P < 0.0001; dependent Student’s *t* test.

HIV-1 efficiently escapes cGAS-mediated innate immune activation in cells such as primary CD4^+^ T cells, macrophages, and dendritic cells (4, 5, 12). The observation that disruption of the uHIV1 chromatin structure results in cGAS activation prompted us to explore whether uHIV-1 DNA may recruit cGAS via its binding to H2A/H2B within viral chromatin leading to its inactivation (2, 3). cGAS KO HeLa cells were stably transduced with Flag-HA-tagged cGAS (cGAS-FHA) or Flag-HA empty vector (EV) (Fig. 3*A*) and then infected with HIV-Luc IN^D116A^ in the presence or absence of the reverse transcriptase inhibitor nevirapine (NVP) for 24 h. cGAS loading on uHIV-1 DNA was investigated by chromatin immunoprecipitation (ChIP) using an anti-HA antibody followed by qPCR analysis using HIV-1-specific primers. α-satellite and GAPDH primers were used as positive and negative controls, respectively (13). Significant enrichment of HIV-1 DNA (Nuc1 and luc) and satellite DNA was observed in cGAS IP from cGAS-FHA HeLa cells but not in those from control (EV) cells (Fig. 3*B* and *C*, Datasets S3 and S4). GAPDH DNA was not significantly enriched. NVP treatment resulted in the loss of HIV-1 DNA but not α-satellite DNA in cGAS IP. Thus, cGAS is loaded onto retrotranscribed uHIV-1 DNA at levels comparable to those on α-satellite DNA. To determine whether cGAS is recruited to uHIV-1 DNA via its binding to histones H2A/H2B, we stably transduced cGAS KO cells with cGAS-FHA harboring two mutations, R236E and R255E (cGAS-FHA R236E R255E), which prevent the nucleosome binding of cGAS (2, 3) (Fig. 3*D*). Notably, the amounts of HIV-1 DNA and α-satellite DNA recovered from cGAS-FHA R236E R255E cells by ChIP were comparable to those recovered from control (EV) cells and significantly lower than those recovered from cGAS-FHA HeLa cells (Fig. 3*E* and *F*, Datasets S5 and S6). Collectively, these results suggest that cGAS is loaded onto uHIV-1 DNA-associated nucleosomes via its binding to histones H2A/H2B.

The discovery that cGAS is primarily tightly bound to nucleosomes, where it is kept inactive, revealed that chromatin is a key regulator of cGAS-mediated innate immune sensing. Notably, uHIV-1 DNA adopts a chromatin structure resembling that of genomic chromatin, where cGAS accumulates (13, 14)—i.e., a nucleosome-dense, H3K9me3-modified chromatin state loaded with the linker histone H1 (15, 16). Thus, we asked whether HIV-1 exploits this regulatory mechanism to evade the cGAS-mediated innate immune response. Disruption of the uHIV-1 chromatin structure by POLE3 KD (9) resulted in cGAS-dependent activation of innate immunity and the establishment of an antiviral state, leading to impaired viral replication. Notably, we found that cGAS was recruited to uHIV-1 DNA via its histone binding domain, leading to its inactivation. Further experiments are required to identify the chromatin changes at the vDNA observed upon POLE3 KD responsible for cGAS-mediated sensing. Our work highlights the hijacking of chromatin-mediated cGAS inactivation as a potential mechanism used by HIV-1 for optimal evasion from innate immunity and suggests that other retroviruses and DNA viruses may exploit this mechanism.

## Materials and Methods

Experimental procedures, cell line, plasmids, and reagents are described in *SI Appendix*. Human primary CD4 T cells were isolated from de-identified blood samples from HIV-uninfected individuals received from “Etablissement Français du Sang.”

## Supplementary Material

Appendix 01 (PDF)

Dataset S01 (XLSX)

Dataset S02 (XLSX)

Dataset S03 (XLSX)

Dataset S04 (XLSX)

Dataset S05 (XLSX)

Dataset S06 (XLSX)

## Data Availability

All study data are included in the article and/or supporting information.
