# Supplementary material for: Unintegrated HIV-1 DNA recruits cGAS via its histone-binding domain to escape innate immunity
Source: Proc Natl Acad Sci U S A. 2025 Mar 11;122(11):e2424465122. doi: 10.1073/pnas.2424465122 (PMC11929445; doi:10.1073/pnas.2424465122)
Supplement: Supplementary file 1 — Appendix 01 (PDF) [file pnas.2424465122.sapp.pdf]

Supporting Information for

Full: Unintegrated HIV-1 DNA recruits cGAS via its histone binding domain to escape innate immunity

Short: Dense chromatin to lure cGAS

Cyprien Jahan[a], Lucie Bonnet-Madin[a], Shinichi Machida[b], Bijan Sobhian[a], Suzie Thenin-Houssier[a,1] and Monsef Benkirane[a]

[1]Corresponding authors
Email: suzie.houssier@igh.cnrs.fr

This PDF file includes:

Supporting text
SI References

Supporting Information Text

Materials and methods

Cells. 293T, HeLa KO cGAS (1), HeLa-P4, HeLa-P4 KO POLE3 (2) were grown in Dulbecco's modified Eagle medium (DMEM) supplemented with 10% FBS, 1 mM sodium pyruvate and antibiotics.

Antibodies. Antibodies for western-blot: Vinculin: sc73614 (SCBT), Tubulin: T5168 (Sigma), cGAS (D1D3G): 15102 (CST), HA.11: 901503 (Biolegend), POLE3: A301-245A (Bethyl). Antibody for ChIP: HA: ab9110 (abcam).

Primary CD4 T cell isolation, culture and activation. De-identified blood samples from HIV uninfected individuals were received from "Etablissement Français du Sang" and human primary CD4 T cells were isolated using MultiMACS^{TM} Cell 24 Separator Plus (Miltenyi Biotec) according to manufacturer's instructions. CD4 T cells were stimulated with 0.5 µg/ml phytohaemagglutinin (PHA, Sigma) and 50 U/ml interleukin 2 (IL-2, Roche) in RPMI-1640 supplemented with 10% heat-inactivated FBS, 1X MEM Non-Essential Amino Acids Solution, 2 mM ultraglutamine, 1 mM sodium pyruvate, 100 U/mL penicillin, and 100 mg/mL streptomycin. Two days later, culture medium was exchanged to medium with 20 U/ml interleukin 2 (IL-2, Roche).

Plasmids. pHIV-Luc IN^{D116A} was provided by S. Emiliani. pNL4-3 was obtained from the NIH AIDS Reagent Program. Plasmids encoding cGAS was provided by N. Laguette. cGAS was cloned in pOZ-Puro-c-ter plasmid to generate cGAS-Flag-HA (cGAS-FHA) plasmid. Mutations R236E and R255E in cGAS-FHA were generated by site directed mutagenesis according to the manufacturer's instructions.

Viral production. Viral stocks (VSV-G pseudotyped HIV-1 replication defective viral particles and NL4.3 replicative virus) were produced in 293T cells using the phosphate calcium transfection method as previsouly described (2).

Overexpression of Flag-HA-tagged-cGAS in HeLa KO cGAS cells. cGAS KO HeLa cells expressing exogenous cGAS-FHA, cGAS R236E R255E-FHA, or FHA-empty vector (EV) protein were generated using the previously described MMLV- based retroviral

constructs (3, 4), containing a bicistronic transcriptional unit allowing expression of a selectable marker (puromycin resistance gene (pOZ-puro)). Cells expressing the different constructs were selected in media supplemented with 1 µg/ml puromycin for 2 days.

Quantification of IFN-related genes in primary CD4^{+} T cells. Primary CD4^{+} T cells were activated with PHA-Il2 and electroporated with 5 µM NT, POLE3 or cGAS siRNA using Amaxa 4D-Nucleofector X Unit (P3 Primary Cell 4D-Nucleofector X Kit (Lonza)). Electroporation setting used for electroporation is Stimulated T cells (P3 solution, EO115 pulse). 48 hrs later, cells were infected with VSV-G pseudotyped HIV-Luc IN^{D116A} for 24 hrs. Cells were harvested and RNAs were extracted using RNeasy Plus mini kit (Qiagen) according to the manufacturer instructions. cDNA synthesis was performed using SuperScript IV enzyme (ThermoFisher Scientific). *IFIT1* and *GAPDH* mRNAs were quantified by qPCR using specific primers (2).

ChIP assay. ChIP assays for cGAS were performed using ChIP-IT High Sensitivity kit ‘Active motif) with some modifications. Briefly, HeLa KO cGAS reconstituted cells (EV, cGAS-FHA and cGAS R236E R255E-FHA) were infected with VSV-G pseudotyped HIV-Luc IN^{D116A} at a MOI of 0.7. After 3 hrs, the cell culture medium was changed. Cells were collected at 24 hpi, and fixed with 1% formaldehyde for 15 min according to manufacturer’s instructions. After stopping fixation and washing in PBS wash buffer, cells were lyzed with lysis buffer 1 (50 mM Hepes-KOH (pH 7.5), 140 mM NaCl, 1mM EDTA, 10% Glycerol, 0.5% NP-40, 0.25% Triton X-100, 1 mM PMSF, and protease inhibitor cocktail) for 10 min on ice. After centrifugation, nuclear pellets were washed with lysis buffer 2 (10 mM Tris-HCl (pH8.0), 200 mM NaCl, and 1 mM EDTA). After centrifugation, nuclei were resuspended with shearing buffer D3 (10 mM Tris-HCl (pH7.4), 1 mM EDTA, 0.1 % SDS, and 1 mM PMSF, and protease inhibitor cocktail), and incubated for 10 min on ice. Chromatin was fragmented by sonication with Covaris S220 evolution, then diluted with same volume of dilution buffer (10 mM Tris-HCl (pH7.4), 300 mM NaCl, 1 mM EDTA, 2% Triton X-100, and 1 mM PMSF, and protease inhibitor cocktail). Immunoprecipitation was performed using 15-20 µg of sonicated chromatin and 3 µg of anti-HA antibody (Abcam ab9110). qPCRs were performed using HIV-1 or genomic specific primers (2).

HIV-1 replication. HIV-1 kinetic of replication was performed as previously described (2). POLE3 KO or NT control cells were treated with 10 µM G140 or DMSO 3 hours before overnight infection with 2 ng P24 of NL4.3 virus (MOI 0.001). Cells were then washed to remove input virus. G140 and DMSO were kept for all the duration of the experiment. Every 3 days, half of culture supernatant was transferred in a new 6 wpf and the other half was collected for p24 ELISA. Cells were trypsinized and equal number of cells were added to the culture supernatant. HIV-1 spreading was measured by assay for p24 concentration in the culture medium using the HIV-1 Gag P24 DuoSet ELISA (Bio-techne, DY7360-05).

SI References

1. M. A. Langereis, H. H. Rabouw, M. Holwerda, L. J. Visser, F. J. M. van Kuppeveld, Knockout of cGAS and STING Rescues Virus Infection of Plasmid DNA-Transfected Cells. *J Virol* **89**, 11169–11173 (2015).
2. S. Thenin-Houssier, *et al.*, POLE3 is a repressor of unintegrated HIV-1 DNA required for efficient virus integration and escape from innate immune sensing. *Sci. Adv.* **9**, eadh3642 (2023).
3. Y. Nakatani, V. Ogryzko, Immunoaffinity purification of mammalian protein complexes. *Methods Enzymol* **370**, 430–444 (2003).
4. D. Kumar, J. L. Shadrach, A. J. Wagers, A. B. Lassar, Id3 is a direct transcriptional target of Pax7 in quiescent satellite cells. *Mol Biol Cell* **20**, 3170–3177 (2009).
